# Supplementary material for: In Vivo Investigation of the Effect of Dietary Acrylamide and Evaluation of Its Clinical Relevance in Colon Cancer
Source: Toxics. 2023 Oct 13;11(10):856. doi: 10.3390/toxics11100856 (PMC10610724; doi:10.3390/toxics11100856)
Supplement: Supplementary file 1 [file toxics-11-00856-s001.zip › Supplementary Figure S1.pptx]

## Slide 1
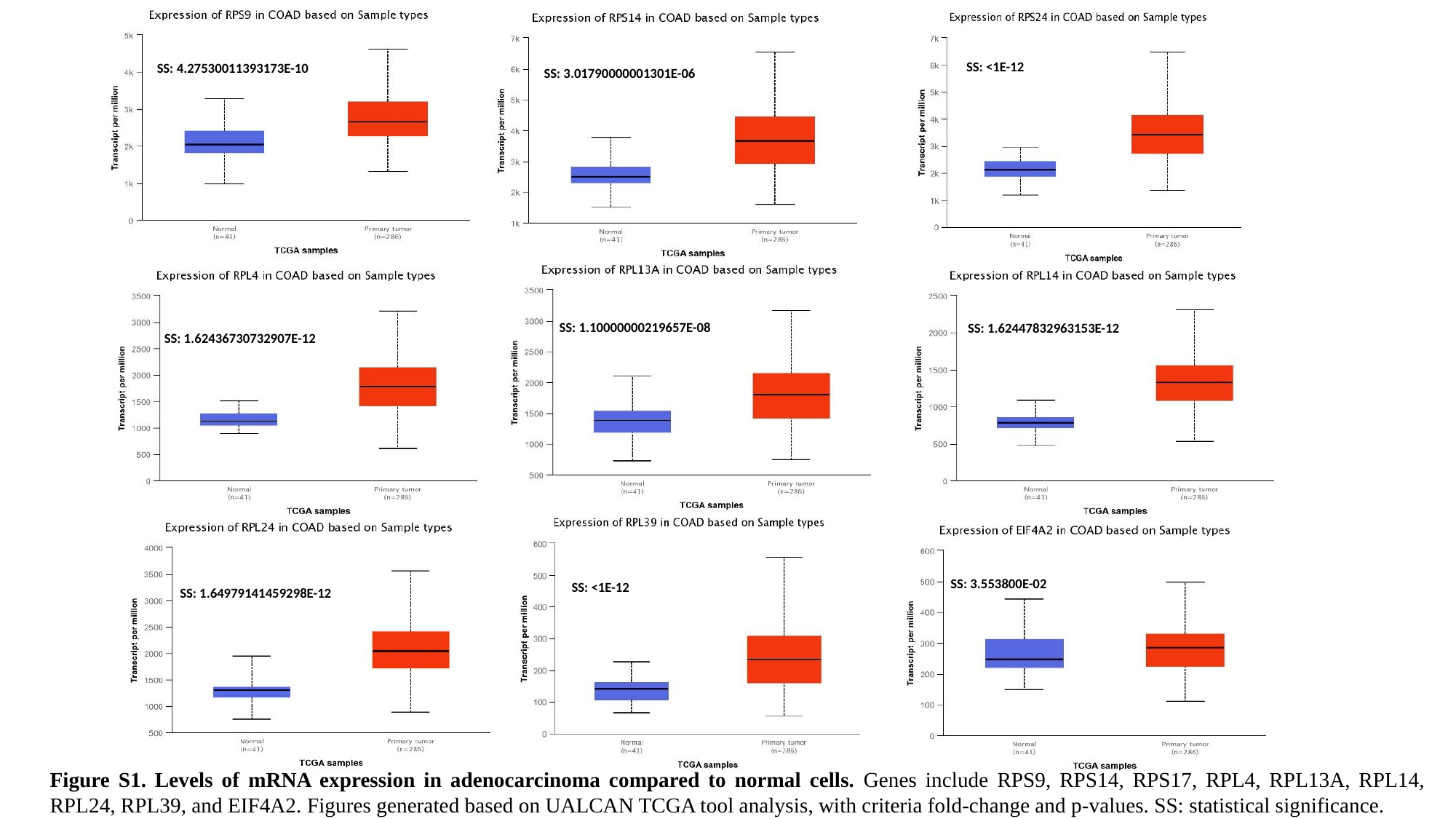

SS: 4.27530011393173E-10
SS: <1E-12
SS: 3.01790000001301E-06
SS: 1.10000000219657E-08
SS: 1.62436730732907E-12
SS: 1.62447832963153E-12
SS: 1.64979141459298E-12
SS: 3.553800E-02
SS: <1E-12
Figure S1. Levels of mRNA expression in adenocarcinoma compared to normal cells. Genes include RPS9, RPS14, RPS17, RPL4, RPL13A, RPL14, RPL24, RPL39, and EIF4A2. Figures generated based on UALCAN TCGA tool analysis, with criteria fold-change and p-values. SS: statistical significance.
